# Longitudinal Association Between Cognition and Depression in Patients With Late-Life Depression: A Cross-Lagged Design Study

**DOI:** 10.3389/fpsyt.2021.577058

**Published:** 2021-10-22

**Authors:** Zhangying Wu, Xiaomei Zhong, Qi Peng, Ben Chen, Min Zhang, Huarong Zhou, Naikeng Mai, Xingxiao Huang, Yuping Ning

**Affiliations:** ^1^Department of Geriatric Psychiatry, The Affiliated Brain Hospital of Guangzhou Medical University (Guangzhou Huiai Hospital), Guangzhou, China; ^2^Department of Neurology, The Affiliated Brain Hospital of Guangzhou Medical University (Guangzhou Huiai Hospital), Guangzhou, China; ^3^The First School of Clinical Medicine, Southern Medical University, Guangzhou, China; ^4^Guangdong Engineering Technology Research Center for Translational Medicine of Mental Disorders, Guangzhou, China

**Keywords:** elderly, depression, cognition, longitudinal, cross-lagged analysis

## Abstract

**Objectives:** Although previous studies have extensively confirmed the cross-sectional relationship between cognitive impairment and depression in depressed elderly patients, the findings of their longitudinal associations are still mixed. The purpose of this study was to explore the two-way causal relationship between depression symptoms and cognition in patients with late-life depression (LLD).

**Methods:** A total of 90 patients with LLD were assessed across two time points (baseline and 1-year follow up) on measures of 3 aspects of cognition and depressive symptoms. The data were then fitted to a structural equation model to examine two cross-lagged effects.

**Results:** Depressive symptoms predicted a decline in executive function (β = 0.864, *p* = 0.049) but not vice versa. Moreover, depressive symptoms were predicted by a decline in scores of working memory test (β = −0.406, *p* = 0.023), respectively. None of the relationships between the two factors was bidirectional.

**Conclusion:** These results provide robust evidence that the relationship between cognition and depressive symptoms is unidirectional. Depressive symptoms may be a risk factor for cognitive decline. The decrease of information processing speed predicts depressive symptoms.

## Introduction

Late-life depression (LLD) is a heterogeneous disorder characterized by a major depressive episode occurring in people 60 years or older, and affects 10–38% of the elderly population (1, 2). It is associated with severe health outcomes including higher risks of mortality and physical disability, or poor quality of life (3, 4). Considering the current rapid growth of the elderly population, it is very important to understand the sequence of depression and cognitive impairment, whether one occurs before the other, adjust the prevention strategy accordingly, and focusing on those with an early onset. However, the interactions between depression and cognitive impairment can be bidirectional, which can make it difficult to distinguish cause and effect.

Many depressed older adults often complain of cognitive symptoms, including mild cognitive impairment or even dementia. Epidemiological findings also suggest that LLD may be a preventable risk for dementia (5). As mentioned above, recent studies have begun to explore the complex relationship between depressive symptoms and cognitive impairment. Among older adults with mild cognitive impairment, those with comorbid depression are 28% more likely to develop dementia than those without depression (6). In fact, not only the occurrence of depressive symptoms, but also the severity of late-life depression has been found to play an important role in the risk of dementia. One of the studies demonstrated that number of depressive symptoms at baseline predicted the development of Alzheimer's disease (AD) within 7 years and each additional depressive symptom increased the risk of AD by 20%l (7). A large number of studies have confirmed that depressive symptoms are the cause of cognitive decline in the elderly and have the role in predicting the risk of dementia. But few studies have explained the causality between the two from a two-way perspective.

On the other hand, some studies suggest that in the process of cognitive decline in the elderly, depressive symptoms may develop. Older individuals with either subjective or objective impairment in executive functioning may respond more poorly to psychopharmacologic interventions, thereby causing relapse and aggravation of depressive symptoms (8). In a 1-year follow-up study, it was found that impairment of some cognitive domains (including verbal memory and processing speed) at baseline were associated with poor response to antidepressant drugs (9). In order to reduce the risk of depression recurrence caused by cognitive decline, some researchers have proposed a combination with antidepressants and cognitive drugs. A study found that escitalopram combined with memantine can effectively control depressive symptoms and improve the condition (10). Otherwise, research on antidepressant drugs combined with donepezil in the treatment of elderly depression found that donepezil does not reduce the recurrence of depressive symptoms (11). Moreover, although donepezil might have potential cognitive benefits, it may also cause more depressive symptoms (11). Therefore, whether the decline in cognitive function can aggravate the symptoms of depression, the results of the current research are inconsistent, and further investigation is needed.

The nature of relationship between depression and cognitive impairment remains controversial. Some studies suggest older adults may develop depressive symptoms in reaction to experiencing cognitive decline, other researchers believe that depression is a risk factor for dementia. There are also theories that depressive symptoms and cognitive decline may be potential neurological symptoms of the degeneration process. These conflicting results may be explained by the methodological differences across studies, such as the age range and clinical characteristics of participants. As mentioned above, research in this area is mostly based on cross-sectional design data, and little is known about the longitudinal causal association between depression and cognition. Similarly, most studies focus on the elderly community and ignore the relationship between the two in clinically symptomatic populations, and the latter group can provide some guidance for clinical treatment.

In recent decades, bidirectional longitudinal studies have been carried out to examine their mutual causality between different variables. Cross-lagged design is one of the types of structural equation modeling. By analyzing the follow-up data collected at two or more time points, the correlation between variables is estimated while controlling for correlations within time points. It is a method that can explain and test the bidirectional nature of a relationship between depression and cognition. Currently it is widely used in various fields, such as exploring the association between depression and loneliness or anxiety in elderly people (12).

In the present study, we aimed to further investigate the relationship between depression and cognitive functioning in the depressive elderly and clarify the direction of influence with the use of cross-lagged panel analysis. Thus, this was the first prospective study to explore the bi-directional relationships between depression and cognition among patients with LLD. We hypothesized that the relation between depression and cognition might be bidirectional, with one of the directions of effect stronger and more stable than the other.

## Methods

### Participants

Ninety patients were recruited from both the outpatient and inpatient units in Department of Geriatric Psychiatry at the Affiliated Brain Hospital of Guangzhou Medical University. The study protocol and the follow-up assessments used in the present study were approved by the ethics committee of the hospital, and all patients signed informed-consent forms before the initial inclusion in the study. The patients met the criteria of major depression above 60 years old, as determined by a structured clinical interview for Axis I DSM-IV (SCID-IV). The exclusion criteria included (1) any other DSM-IV Axis I disorders; (2) severe or unstable medical conditions; (3) neurological disorders such as stroke, delirium, Parkinson's disease, brain tumors; (4) conditions that might cause depression, such as hypothyroidism or drugs (glucocorticoids and interferon); (5) a current history of alcohol use to a degree of alcohol dependence; (6) head injury with loss of consciousness>30 min; (7) poor vision or failure to complete the examination; (8) A pre-existing diagnosis of dementia was an exclusion criterion.

### Measures

Demographic information (sex, age, education, age of onset, and course of disease) was collected at baseline only. Measures of depressive symptoms and cognition were collected at baseline (T1) and 1-year follow-up (T2).

Depressive symptoms were assessed with 17-item Hamilton Rating Scale for Depression (HAMD). The HAMD consisted of 17 items. Higher scores on HAMD corresponded to worse outcomes and severity of depression. The HAMD assessment was completed by two trained professional psychiatrists who passed the consistency assessment.

### Cognitive Measures

Participants completed a battery of 3 cognitive measures, including: (1) memory: the scores of Auditory Verbal Learning Test at 20 min(AVLT-5); (2) executive function: times of Trail Making Test(TMT-B); (3) information processing function: scores of Working Memory Test (WMT). Except for the scores of TMT-B, the higher scores indicate better cognitive performance in other 3 subscales. Cognitive assessments were completed by two trained research assistants who passed the consistency assessment.

### Data Analyses

First, baseline characteristics of the study sample were presented using descriptive statistics, including frequencies and percentages for categorical variables (such as sex and education level), and means and standard deviations for continuous variables (depressive symptoms and cognitive performance).

Second, Pearson product-moment correlation was used to analyze the inter-correlations among depression and cognition at two time points.

Third, a cross-lagged panel analysis was carried out via structural equation modeling to examine the relationship between cognition and depression. Age, education, age of onset, course of disease were included as covariates in the model. Missing data were addressed using full information maximum likelihood. Assumptions of linear modeling were violated for one variable, namely depression at follow-up. The variable was too skewed for a transformation so the analysis was bootstrapped using 2000 bootstrap draws. Model fit indies were determined by comparative fit index (CFI), Tucker-Lewis index, root mean square error of approximation (RMSEA) and standardized root mean square residual (SRMR). RMSEA value of <0.05 were considered a very good fit. RMSEA values of <0.05 were considered good. SRMR values <0.08 are indicative of an acceptable model. Cross-lagged analysis was carried using AMOS, the statistical program provided by IBM 22.0 and the package for structural equation modeling. Correlations between variables were examined using Pearson correlation coefficients. Statistical significance was set at *p* < 0.05.

## Results

Table 1 shows the baseline characteristics of the study sample. The study sample comprised 90 patients with LLD aged 60–88 years old (mean age = 68.67 years, SD = 7.565 years; 76.7% are female). Some of them had a sixth-grade education or below (28.4%). The depressive symptoms at baseline was significantly higher than that 1-year follow up (*t* = −0.479, *p* = 0.633). None of the scores in different aspects in cognition was significantly different between T1 and T2.

**Table 1:** Characteristics of participants at T1 and T2.

	**Participants with data at T1**	**Participants with data at T2**	***t*(df)**	***P***
	**Means (SD)/frequency (*N*%)**	**Means (SD)/frequency (*N*%)**		
**Sex**
Male	21 (23.3)	-	-	-
Female	69 (76.7)	-	-	-
Age, years	68.67 (7.565)			
60–69	65 (63.7)	-	-	-
70–79	30 (29.4)	-	-	-
≥80	7 (6.9)	-	-	-
Years of Education	8.82 (4.23)			
≤ 6	29 (28.4)	-	-	-
7–12	45 (44.1)	-	-	-
≥12	28 (27.5)	-	-	-
**Source of patient**
Hospitalized	2			
Outpatient	88			
Hypertension	26 (28.9)			
Diabetes	10 (11.1)	-	-	-
Hyperlipidemia	35 (38.9)	41 (45.6)	61.452 (1)	0.001^*^
**Depressive symptoms**
HAMD	6.67 (5.306)	6.93 (5.971)	−0.479 (89)	0.633
**Scores of HAMD**
≤ 7	57 (63.3)	54 (60)	10.293 (1)	0.001^*^
8–16	27 (30)	29 (32.2)		
≥17	6 (6.7)	7 (7.8)		
Percentage of MDD	6 (6.7)	7 (7.8)		
**Cognition**
Percentage of SCD	2 (2.2)	1 (1.1)		
Percentage of MCI	69 (76.7)	74 (82.2)	58.832 (1)	0.000^*^
MMSE	24.12 (4.261)	24.09 (4.730)	0.095 (89)	0.925
AVLT-N5	4.95 (3.125)	4.68 (3.446)	1.064 (86)	0.291
DST-N2	5.05 (1.613)	5.08 (2.024)	−0.161 (86)	0.873
WMT	3.55 (2.604)	3.93 (2.463)	−1.455 (83)	0.149
TMT-B	82.87 (3.872)	88.41 (3.915)	−1.688(84)	0.095

* *P < 0.05; MDD, major depressive episode; SCD, subjective cognitive disorder; MCI, mild cognitive impairment*.

Table 2 describes the correlations between depression and cognition at T1 and T2. The correlations between depressive symptoms at T1 and T2, depressive symptoms at T2 and working memory scores at T2, depressive symptoms at T2 and memory scores at T2 reached significance. All the correlations between different cognitive scales were also significant.

**Table 2:** Descriptive statistics and inter-correlations among depression and cognition across T1 and T2.

**Variable**	**1**	**2**	**3**	**4**	**5**	**6**	**7**	**8**
1.T1D	1							
2.T2D	0.566^**^	1						
3.T1M	−0.41	−0.183	1					
4.T2M	−0.098	−0.223^*^	0.733^**^	1				
5.T1I	−0.053	−0.197	0.408^**^	0.427^**^	1			
6.T2I	−0.204	−0.232^*^	0.427^**^	0.439^**^	0.553^**^	1		
7.T1E	−0.002	−0.033	−0.562^**^	−0.566^**^	−0.431^**^	−0.449^**^	1	
8.T2E	−0.096	0.183	−0.648^**^	−0.664^**^	−0.439^**^	−0.482^**^	0.645^**^	1

* *P < 0.05;*

** *P < 0.01*.

*T1, time 1; T2, time 2; M, memory; I, information processing speed; E, executive function*.

### Temporal Relationship Between Depression and Cognition

The cross-lagged path analysis was run in one model that included depression at both time points and all different cognitive variables. Table 3 describes the cross-lagged effects for depression and cognition in 2 time points. Results are illustrated in 3 separate diagrams.

**Table 3:** Cross-lagged effects for depression and cognition.

**Effect**	***P***	**SE**	**95%CI**
**Cross-lagged**
T1D→ T2M	0.381	0.046	−0.119, 0.037
T1D→ T2W	0.350	−0.094	−0.250, 0.071
T1D→ T2E	0.049^*^	0.864	0.151, 1.637
T1M→ T2D	0.119	0.162	−0.616, 0.019
T1W→ T2D	0.023^*^	−0.177	−0.307, −0.049
T1E→ T2D	0.484	−0.009	−0.028, 0.011

* *P < 0.05; T1, time 1; T2, time 2; M, memory; I, information processing speed; E, executive function; W, working memory test; D, depression*.

**Figure 1:**
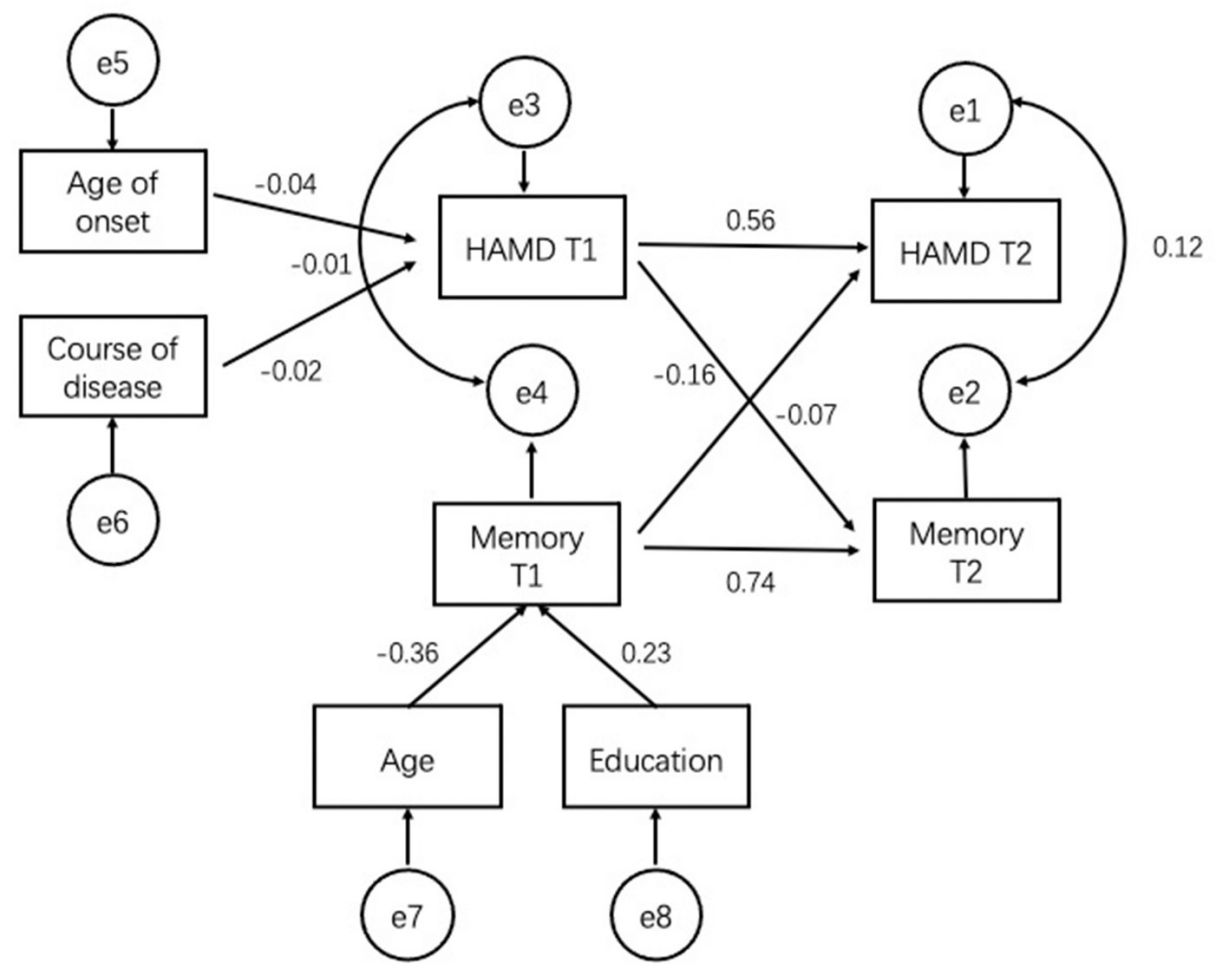
Robust maximum-likelihood estimation of the cross-lagged model with age, education, age of onset, and course of disease included as covariates. Figure in parentheses represent standard errors of the estimates. Straight line represent regression paths. Curve lines represent residual covariance. Memory: scores of AVLT-N5. T1, time 1; T2, time 2.

With regard to cross-lagged effects, there was no significant cross-lagged effect in memory test of model 1 (Figure 1).

**Figure 2:**
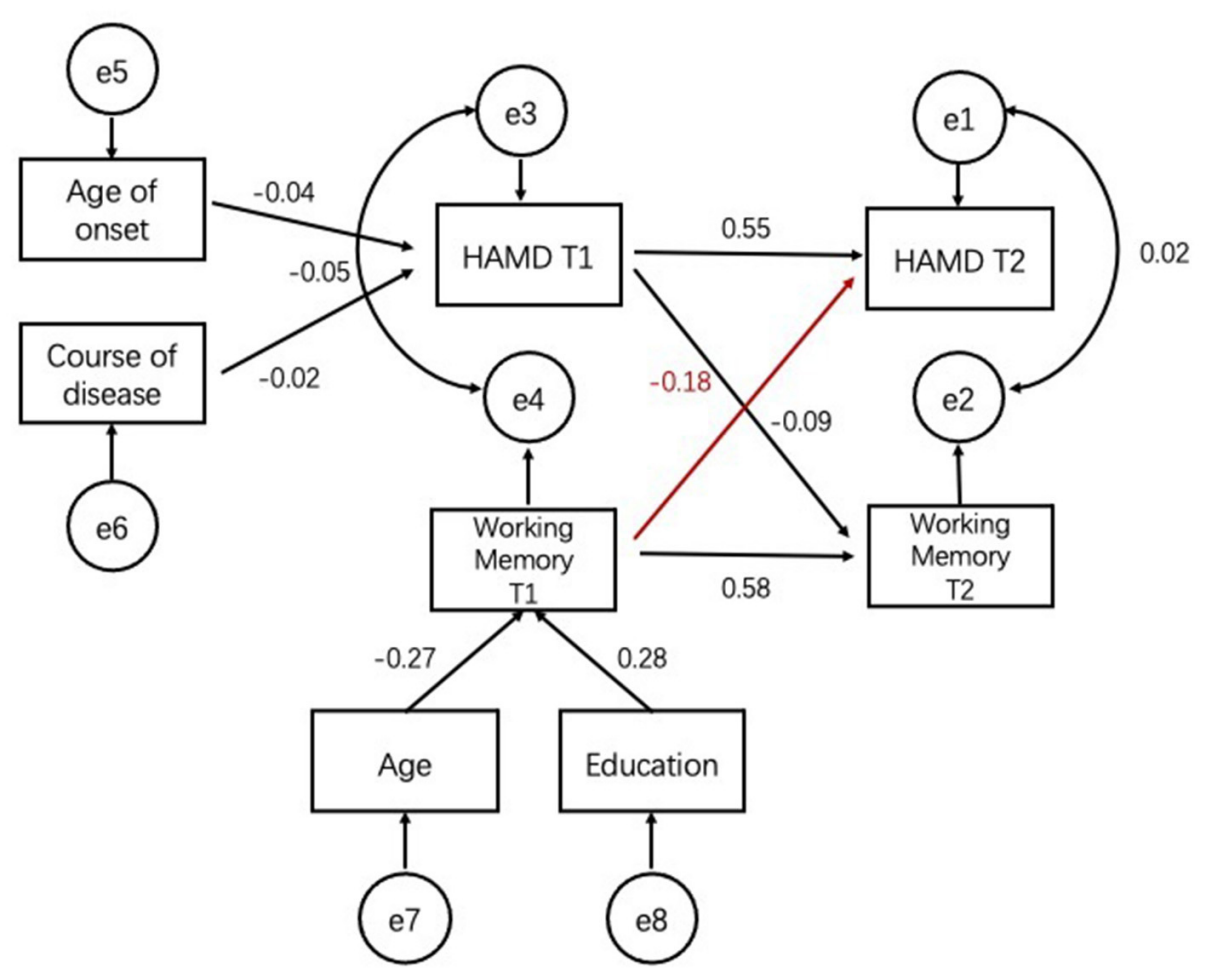
Robust maximum-likelihood estimation of the cross-lagged model with age, education, age of onset, and course of disease included as covariates. Figure in parentheses represent standard errors of the estimates. Straight line represent regression paths. Curve lines represent residual covariance. Working Memory: scores of Working Memory Test. T1, time 1; T2, time 2.

Figure 2 shows the cross-lagged relationship between depressive symptoms and scores of working memory test between two time points. There was a significant negative relationship between depression scores at T2 of working memory (β = −0.406, *p* = 0.023) at T1 but no significant association for the reciprocal relationship (β = −0.046, *p* = 0.350) (Figure 2).

**Figure 3:**
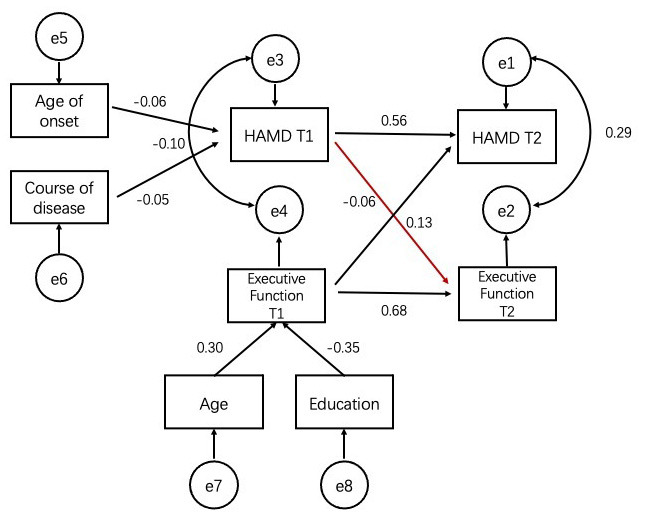
Robust maximum-likelihood estimation of the cross-lagged model with age, education, age of onset and course of disease included as covariates. Figure in parentheses represent standard errors of the estimates. Straight line represent regression paths. Curve lines represent residual covariance. Executive function: scores of TMT-B. T1, time 1; T2, time 2.

Figure 3 shows the cross-lagged relationship between depression and TMT-B. There was a significant positive relationship between depression at T1 and scores of executive function test (β = 0.864, *p* = 0.049) at T2 but no significant association for the reciprocal relationship (β = −0.009, *p* = 0.484) (Figure 3).

## Discussion

In the present study, cross-lagged analysis found that depressive symptoms predicted a decrease in executive function otherwise depressive symptoms were predicted by lower working memory function. The data did not support our previous hypothesis of reciprocal relationship. None of the relationships between the two was bidirectional in depressed elderly people. To the best of the authors' knowledge, this is the first study to find evidence that the bidirectional relationship between cognitive function and depressive symptoms in LLD.

One of the key findings of this study was higher depressive symptomatology predicted poorer cognitive performance on executive function indicating that depressive symptoms could either be a risk factor of cognitive decline. This finding was in agreement with results from hypothesis demonstrating the “risk factor hypothesis” (13). Previous studies have found executive impairment in patients with LLD, but most of them are cross-section data (14, 15). This study found that from the perspective of a longitudinal follow-up, the severity of depression can predict the level of executive function in LLD to some extent. Our results were in contrast to some previous evidence for relationship between depression and executive function. They regarded that depression is a psychological reaction to the subjective experience of cognitive decline. Regrettably we could not find the bidirectional relationship of executive function. Neuroimaging research have been revealed that frontal lobe mediated deficits in executive function was atrophy or dysfunctional in LLD patients (16). We believed that impaired executive function in LLD patients is more related to the variation of neurophysiological anatomy rather than a psychological response.

Executive function is one of the aspects of cognition that allows individuals to focus, plan, organize, and perform task. Unlike other domains of cognitive function, impairments of executive function are difficult for an individual to independently compensate. Therefore, in patients' daily life experiences, the slight decline in executive function may be the most obvious than in other cognitive function areas (17).

Depressive symptoms at follow-up were predicted by lower levers of information processing speed at baseline but not attention and memory. This gives partial support for the “psychological reaction” hypothesis. In keeping with the results of previous studies, which demonstrated that slowing of information processing speed persisted even after depression remitted (18). Information processing speed has been suggested to mediate all other cognitive impairments (19, 20). As our results showed, the persistence of impairment of information processing speed raises the possibility that there is an underlying neurobiological mechanism in LLD patients. Although we found that depression did not predict subsequent information processing speed over 1-year follow up. Nevertheless, we speculate that the influence of depression on information processing speed may not be apparent within the relatively shorter study period and most of the patients were in a remitted state.

The strength of the current study is in its use of a clinical population cohort sample allowing the detection of small effects and the use of cross-lag analysis to simultaneously examine bidirectional relationships between depressive symptoms and cognition. The present study has several limitations. Firstly, the sample size was relatively small thus the current study may not have enough power in detecting cross-lagged effects. Patients with LLD often have symptoms of anxiety. It is impossible to completely eliminate the impact of anxiety on cognitive function and necessary to add the evaluation and observation of anxiety symptoms to the future cognitive function research of LLD. Secondly, most patients were in a state of remission and cannot fully reflect the temporal relationship between different levels of depression and cognitive function. Further studies should focus on this issue to include more subjects with recurrent LLD. Thirdly, this study included data from the two time points of baseline and one-year follow-up, and could not fully track the relationship between depressive symptoms and long-term longitudinal changes in cognitive function. Although more time points that span over a few years will provide more information about the longitudinal relationship between depression and cognition, our findings are still the basis for future studies to consider setting repeating evaluation intervals over a longer period of time.

Notwithstanding these limitations, the current study is the first to explore the longitudinal associations between cognition and depression in LLD patients using a cross-lagged panel method. Although we did not find a bidirectional relationship between depressive symptoms and cognition, our results suggested that baseline depression predicted subsequent executive function, otherwise, depression was predicted by information processing speed. These results suggest that for elderly patients with depression, the complex relationship between cognition and depressive symptoms. Further, the combination of antidepressant therapy and nootropic therapy may help promote the overall recovery of the patient's condition.

## Data Availability Statement

The raw data supporting the conclusions of this article will be made available by the authors, without undue reservation.

## Ethics Statement

The studies involving human participants were reviewed and approved by Ethics Committee of the Affiliated Brain Hospital of Guangzhou Medical University. The patients/participants provided their written informed consent to participate in this study.

## Author Contributions

ZW: conception and design of this study, acquisition and analysis of the neuropsychological data, and drafting the manuscript and final approval of the version to be published. YN: conception and design of this study, revising the article, and final approval of the version to be published. XZ: revising the article and final approval of the version to be published. QP and MZ: acquisition of the clinical and neuropsychological data and final approval of the version to be published. BC: acquisition and revising of the clinical data and final approval of the version to be published. NM and HZ: acquisition of the clinical data and final approval of the version to be published. XH: acquisition and of neuropsychological data and final approval of the version to be published. All authors contributed to the article and approved the submitted version.

## Funding

This study was supported by a grant from the National Natural Science Foundation of China (No. 82171533), the Key Laboratory for Innovation Platform Plan, the Science and Technology Program of Guangzhou, China, and the Science and Technology Plan Project of Guangdong Province (No. 2019B030316001).

## Conflict of Interest

The authors declare that the research was conducted in the absence of any commercial or financial relationships that could be construed as a potential conflict of interest.

## Publisher's Note

All claims expressed in this article are solely those of the authors and do not necessarily represent those of their affiliated organizations, or those of the publisher, the editors and the reviewers. Any product that may be evaluated in this article, or claim that may be made by its manufacturer, is not guaranteed or endorsed by the publisher.
